# A brain connectivity characterization of children with different levels of mathematical achievement based on graph metrics

**DOI:** 10.1371/journal.pone.0227613

**Published:** 2020-01-17

**Authors:** Sulema Torres-Ramos, Ricardo A. Salido-Ruiz, Aurora Espinoza-Valdez, Fabiola R. Gómez-Velázquez, Andrés A. González-Garrido, Israel Román-Godínez

**Affiliations:** 1 Departamento de Ciencias Computacionales, Universidad de Guadalajara, Guadalajara, México; 2 Instituto de Neurociencias, Universidad de Guadalajara, Guadalajara, México; Georgia Institute of Technology, UNITED STATES

## Abstract

Recent studies aiming to facilitate mathematical skill development in primary school children have explored the electrophysiological characteristics associated with different levels of arithmetic achievement. The present work introduces an alternative EEG signal characterization using graph metrics and, based on such features, a classification analysis using a decision tree model. This proposal aims to identify group differences in brain connectivity networks with respect to mathematical skills in elementary school children. The methods of analysis utilized were signal-processing (EEG artifact removal, Laplacian filtering, and magnitude square coherence measurement) and the characterization (Graph metrics) and classification (Decision Tree) of EEG signals recorded during performance of a numerical comparison task. Our results suggest that the analysis of quantitative EEG frequency-band parameters can be used successfully to discriminate several levels of arithmetic achievement. Specifically, the most significant results showed an accuracy of 80.00% (*α* band), 78.33% (*δ* band), and 76.67% (*θ* band) in differentiating high-skilled participants from low-skilled ones, averaged-skilled subjects from all others, and averaged-skilled participants from low-skilled ones, respectively. The use of a decision tree tool during the classification stage allows the identification of several brain areas that seem to be more specialized in numerical processing.

## Introduction

Cerebral functional processes can be evaluated through data acquired using different techniques. In this context, data obtained through neuroimaging methods, like fMRI and PET, provide information about brain activity with high spatial resolution, while electrophysiological techniques provide meaningful high-temporal resolution brain data. Due to their non-invasiveness, low cost, real-time analysis capability, and compatibility with portable devices [1], electroencephalographic (EEG) studies have emerged as a particularly advantageous and commonly-employed technique.

EEG methods have been used increasingly to study developmental processes, such as math learning, particularly in relation to arithmetic. Since this is a very complex process involving a wide range of cognitive abilities, current educational and clinical parameters used for diagnosis and definitions of dyscalculia, which is a specific learning disability that affects the acquisition of arithmetic skills [2], are both highly-variable and subjective. Thus, searching for specific and sensitive biological markers (biomarkers) has been a goal of recent research.

Specifically, EEG coherence measures, which represent the degree of association or coupling of frequency spectra between two different time series, have been considered a reflection of integrated cortical functions [3]. EEG coherence measures also represent the functional association between different brain regions [4] and have, therefore, been used to evaluate the neurofunctional substrates underlying the learning of specific math concepts [5], arithmetic operations [6, 7], reverse counting [8], proportional reasoning [9], identification of digit sequences [10], and numerical comparison [11] in different populations.

EEG data analysis, however, presents several difficulties due to its complexity and variability, susceptibility to noise, and the need to explore the data at different levels (high dimensionality) [12]. As a result, many efforts have been made to address neuroscience problems from mathematical and computational perspectives. In general, most of them share a common workflow that involves an experimental sampling, pre-processing and characterization, and the construction of a machine-learning model that captures the correlation that exists between certain features and the mathematical task.

Regarding cognitive function prediction, numerous reports have predicted different mental mathematical tasks [13–16]. Such experiments may be performed by a single participant (intra-participant) or group (cross-participants). Specifically, analyzing EEG signals using Decision Tree models (DT), the authors of [17] showed that it is possible to identify EEG signal patterns that successfully distinguish between alcoholic individuals and non-alcoholic people. In addition, the authors of [18] propose using DT in feature selection; that is, selecting an EEG channel for Brain-Computer Interface applications. This has been shown to produce competitive classification performances and also has the advantage that during the learning phase the DT model selects the important electrodes involved in the classification stage and depicts them by a decision tree.

Alternatively, there is a network-based approach related to the cognitive function of the brain, which holds that it is possible to explore a subject’s underlying mental function and behavioral performance by identifying changes in the functional connectivity pattern induced by a task (i.e., network-based prediction of cognitive processing) [19]. This approach is based on the application of graph theory, where a graph is a mathematical object consisting of vertices and edges representing elements and connections, respectively [20, 21].

Despite all these efforts to clarify the electrophysiological characteristics that distinguish mathematical processing, this issue remains unresolved. In this context, the first objective of the present study was to propose an alternative characterization of EEG signals based on several graph metrics. We believe that quantifying brain connectivity information through graph metrics will perform well, since EEG signals feature extractors and previous work has demonstrated the importance of network-based analysis [19–21]. Second, the proposed EEG signal characterization, along with a machine-learning model, opens the possibility of doing both; that is, predicting children’s levels of mathematical achievement using biological markers, and identifying the brain functional association patterns that allow such predictions. Even though the aforementioned machine-learning models present good performances [13–16], visualizing and interpreting the patterns are difficult; hence, extracting additional information from the data is complicated or requires additional tools. As an alternative, the present work proposes using a DT model in light of its advantage of being easily transformed into decision rules that, in turn, simplify interpretation [22].

The study is divided into two stages: first, the physiological signal-processing which involved reducing artifacts and volume conduction effects, calculating the coherence coefficient, and performing the graph metric-based feature extraction. The second stage consisted in detecting the outlier participants and machine-learning modeling steps. For the machine-learning modeling we used a decision tree model, under the hypothesis that implementing these computational and mathematical tools could contribute to identifying electrophysiological differences between groups of children with distinct math skills.

## Materials and methods

The following sections discuss the participants and details of data acquisition. After that, our proposal is presented, organized in two stages: physiological signal-processing and machine-learning processing.

### Participants’ data

Data were obtained from [11], which includes a total of 57 children aged 8-11 years. This sample, including both girls and boys, was selected from a pool of 441 third-graders from six public and three private elementary schools. Participants were categorized into three groups according to their performance on the math subsection of the Wide Range Achievement Test, 4^th^ edition [23]: Low Achievement (LA), those within the lowest 15^th^ percentile (19 participants); Average Achievement (AA), between percentiles 40 and 60 (20 participants); and High Achievement (HA), within the highest 15 percentiles (18 participants). Table 1 shows the participants grouped in their respective achievement level distribution as well as their age range.

**Table 1:** Participant distribution by level of achievement and age range.

Achievement level	Total	Age range (years)
HA	18	8.6 − 9.9
AA	20	8.2 − 9.9
LA	19	8.3 − 10.8
Total	57	-

The children’s teachers confirmed the math skill level assigned according to the WRAT-4 results. All participants were matched according to their educational environment, such that each LA subject was paired with a child from the same school with AA and HA. This split criterion has been used previously to explore different aspects of neurodevelopment in children with distinct math abilities [11, 24–26]. Children with antecedents of neurological, psychiatric or behavioral disorders were not included in the final pool of participants, none of whom had ever repeated a school grade. Prior to the study, the parents provided written informed consent. All procedures involved were approved by the ethics committee of the Institute of Neuroscience (Registration number: ET112012-128).

All participants were instructed to perform a symbolic numerical comparison task in which Arabic numerals were presented in pairs. They were asked to determine which of the two represented the larger quantity. The stimuli consisted of 112 pairs of Arabic numerals (in 54-point Arial font) with values ranging from 5 to 34. These numerals were presented in several pairs (5 − 10; 7 − 14; 10 − 20; 11 − 22; 13 − 26; 14 − 28; 17 − 34; 6 − 8; 9 − 12; 12 − 16; 15 − 20; 18 − 24; 21 − 28, and 24 − 32). The numbers were presented on a 17-inch LCD monitor (screen resolution = 1280 × 1024; frame rate = 60 Hz). Participants were seated comfortably, 60 cm from a computer screen, in a dimly-lit, sound-attenuated room. They had to respond according to the position in which the larger number appeared on the screen (left or right) by pressing a button with the corresponding index finger (left or right) as fast as possible. The stimuli appeared in two separate blocks, in a counterbalanced order across participants.

Each trial began with the display of a fixation cross (400 ms), followed by the pair of numerical values (1000 ms), and then a gray screen (400 ms); which set a maximum response time of 1400 ms. The children were asked to focus on the fixation cross to prevent eye movement. Stimulus presentation and response registration were performed using the MINDTRACER software package (Neuronic systems, Inc.). All EEG signals were recorded simultaneously.

### Recording

Electrophysiological (EEG) activity was recorded from 19 scalp electrode sites (C3, C4, CZ, F3, F4, F7, F8, FZ, Fp1, Fp2, O1, O2, P3, P4, PZ, T3, T4, T5 and T6) using an Electro-Cap (Electrocap Inc., Eaton, Ohio). Electrode sites were referenced to linked-ear lobes. Inter-electrode impedances were less than 5 kilo-ohms at 30 Hz. EEG signals were amplified at a band-pass of 0.5-30 Hz (3dB cutoff points of 6dB/octave roll-off curves) with a sampling period of 5 ms (Fs = 200 samples/s) in a MEDICID-04 system (Neuronic Systems, Inc.).

### Physiological signal-processing

In this stage, artifacts are removed from the EEG signals, a region of interest is selected, and a spatial filter is applied. These steps ensure the correct calculation of the coherence between electrodes in different frequency bands.

#### Artifact removal

An important issue in EEG protocols is that artifacts from different sources can contaminate the measured signals. These undesirable signals make EEG interpretation more difficult and alter such quantitative analysis methods as coherence. Ocular and muscular artifacts are the most common ones seen in EEGs [27]. Previous studies have proposed an automated method for the detection and removal of ocular and myogenic artifacts for multi-channel EEG recording [28], while other authors have focused only on removing ocular artifacts by combining ICA and regression methods [29]. An alternative to this is to remove artifacts by manually selecting and removing the independent components that correspond to artifacts, followed by a free artifact EEG reconstruction [30–32]. Most of the artifact removal techniques used in EEG signals include signal decomposition, such as Blind Source Separation (BSS) or Independent Component Analysis (ICA) [31, 33, 34].

In this study, we applied an algorithm to decompose the 19-channel artifact-mixed EEG signals into independent components using FastICA [35]. The estimated independent components that the expert judged to be muscular and ocular artifacts were removed, then a signal reconstruction was performed to obtain an EEG signal that was free from muscular and ocular artifacts [31]. This artifact removal implementation was performed using Python 3.7 and a Python toolkit for data-processing and machine-learning, called scikit-learn, using a function called *FastICA* from the package *sklearn.decomposition* [36].

**Fig 1:**
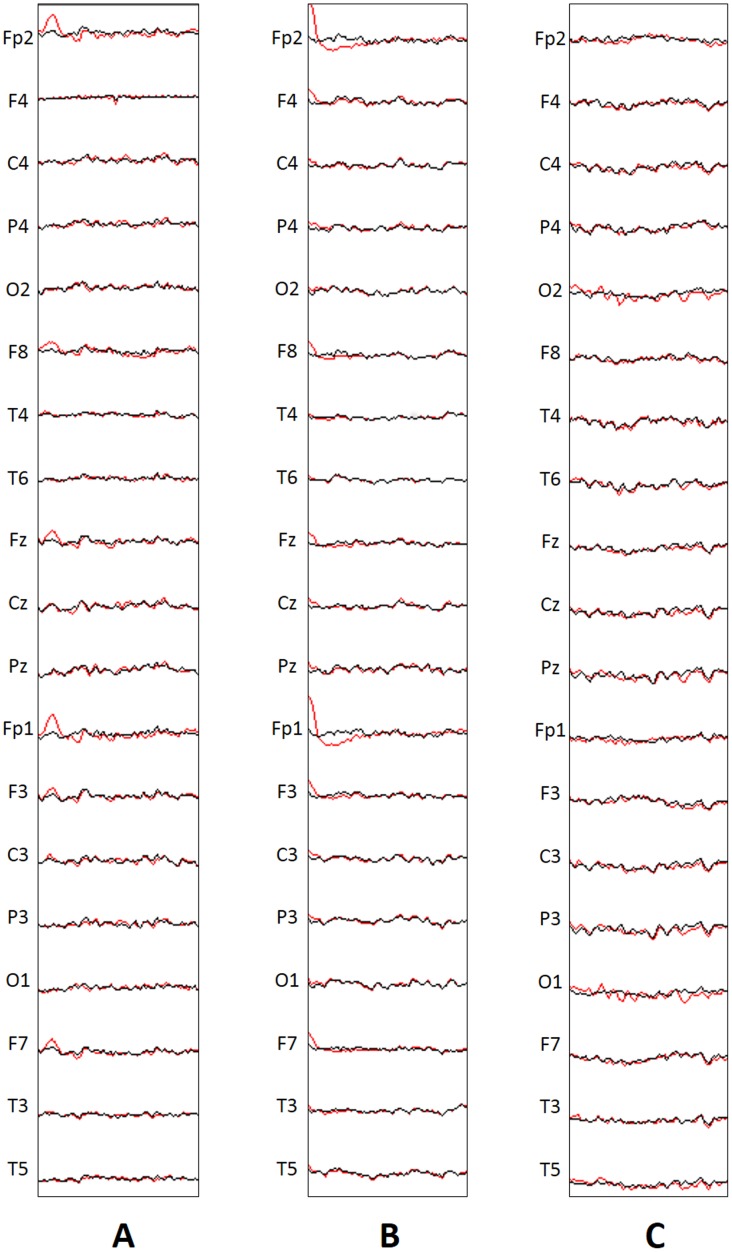
Suppression of muscular and ocular artifacts for selected subjects with HA (A), AA (B) and LA (C). One-second window lengths of raw EEGs with artifacts (red) and corrected EEG (black) are shown.

Fig 1 shows some ocular artifacts in the raw EEG signal at Fp2 and Fp1 electrode channels in red, and their suppression in black for selected subjects in HA (**A**), AA (**B**) and LA (**C**).

#### Window selection

The experimental task consisted of 112 trials and the group of participants with lower behavioral performance (LA) had an average of 73 correct responses (Mean: 73.7, SD: 7.1).

After performing an artifact removal technique (see S1 File) for each participant’s EEG recording, 1000-ms time windows –from 100 ms before to 900 ms after stimulus onset– corresponding to artifact-free, correct behavioral responses were selected (i.e. 57 similar epochs, each of 1000 ms). In all groups, the same number of EEG time windows was selected per individual for further analysis.

#### Surface Laplacian

**Fig 2:**
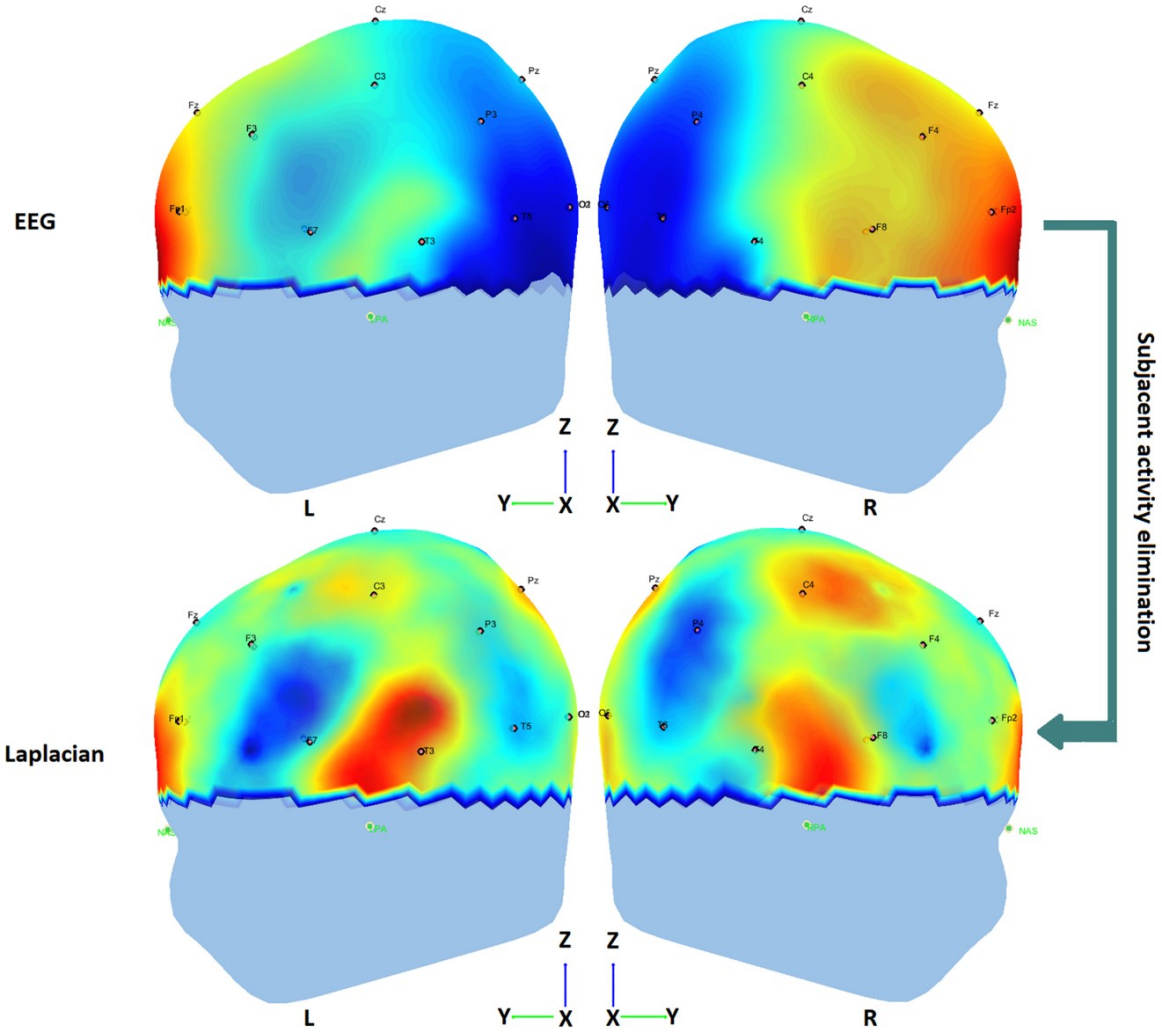
Graphic representation of the effect of Laplacian spatial filtering in one high-achievement subject during task onset.

The ability to distinguish small spatial details from EEG (or spatial resolution of EEG) is limited by the volume conduction of current from the brain through the scalp. This is due to the spatial smearing effect of the poorly conductive-skull layer. Studies have demonstrated that volume conduction can elevate EEG coherence at all frequencies for moderately-separated (< 10 cm) electrodes [37]. The EEG Surface Laplacian is a spatial filter that allows the estimation of current densities entering (or exiting from) the scalp through the skull [38]. Surface Laplacian is the second spatial derivative of the scalp potential at two surface coordinates tangent to the local scalp [39]. A first improvement in the resolution of this technique was presented in [40], where more elaborated Surface Laplacian techniques (i.e., fitting the instantaneous potential values employing interpolation by spline function techniques) were used to improve the estimation of scalp potential topographies [40]. However, more recent improvements to these estimations are presented in [38, 41] and used in this work. Since Laplacian isolates the source activity under each electrode that is distinct from the surrounding tissue, it is strongly recommended to apply Laplacian EEG methods, prior to EEG coherence estimations. In other words, this pre-processing step will allow us to ensure that such a measure can be directly related to the coherence between the sources we are considering. Therefore, the Surface Laplacian technique was applied to the signals that resulted from the previous step, (see Fig 2). We implemented a Spline Surface Laplacian with realistic geometry (SSL-Geo), a new algorithm available in *ssltool*, a free toolbox for Matlab^®^ for bioelectromagnetic data visualization, Surface Laplacian calculation and modeling that can compute Surface Laplacians directly on triangular meshes and gives better results than spherical algorithms [38, 41]. In this context, a three-layer realistic head geometry model, originally described for adults [38], was built in order to implement the Surface Laplacian solution. Due to the age of our participants, we decided to tailor the final spatial sampling by adjusting our calculations to the averaged coordinates of the sensor position files established in the EGI 3-D model for children aged 2-9, and 9-18 years, respectively, in each electrode location. The realistic head geometry model consisted of an innermost brain structure layer surrounded by two realistic-geometry structures for the skull and scalp. In Fig 2 the potentials of EEG recordings in one of the subjects look somewhat diffuse over the scalp, showing some apparently synchronized regions that become more focused and less synchronized after applying the Laplacian spatial filter, perhaps due to the subjacent activity suppression.

#### Coherence correlation measurement

Coherence is a function that measures the statistical interrelation between two signals in the frequency domain. Coherence coefficient values are normalized between 0 and 1 [42–45]. In order to determine the connectivity between electrodes, the magnitude square coherence was calculated in the frequency domain *ω*, defined by Eq (1):

$$\begin{array}{c} \phi_{ij}\left(\omega \right)=\frac{{\left[S_{ij}\left(\omega \right)\right]}^{2}}{S_{ii}\left(\omega \right)S_{jj}\left(\omega \right)},\;with\;i,j\in \left\{1,2,..,n\right\}, \end{array}$$ (1)

where *ϕ*_*ij*_ is the coherence spectrum matrix, *i* and *j* are the electrodes for which coherence is calculated, *n* is the total number of electrodes, *S*_*ii*_ and *S*_*jj*_ are auto-spectrum densities of the time series, and *S*_*ij*_ is the cross-spectrum density from both signals. This gives the coherence of all the electrodes for all frequencies up to *Fs*/2 (Nyquist Frequency), where *Fs* is the sampling frequency of the signals. A coherence coefficient matrix can be obtained from the coherence spectrum matrix that results from Eq (1) by integrating all the coherence values in the frequency band of interest or for the entire frequency content:

$$\begin{array}{c} M_{ij}=\frac{2}{Fs}\int_{ll}^{ul}\phi_{ij}(\omega )d\omega ,\;where\;i,j\in \left\{1,2,...,n\right\}, \end{array}$$ (2)

where *ll* and *ul* are the lower and upper limits of the integral, respectively. Eq (2) provides a general interrelationship between the electrodes among the entire frequency, or a specific frequency band of the EEG signals. These interrelationships form a symmetric matrix **M** of dimensions *n* × *n*.

**Fig 3:**
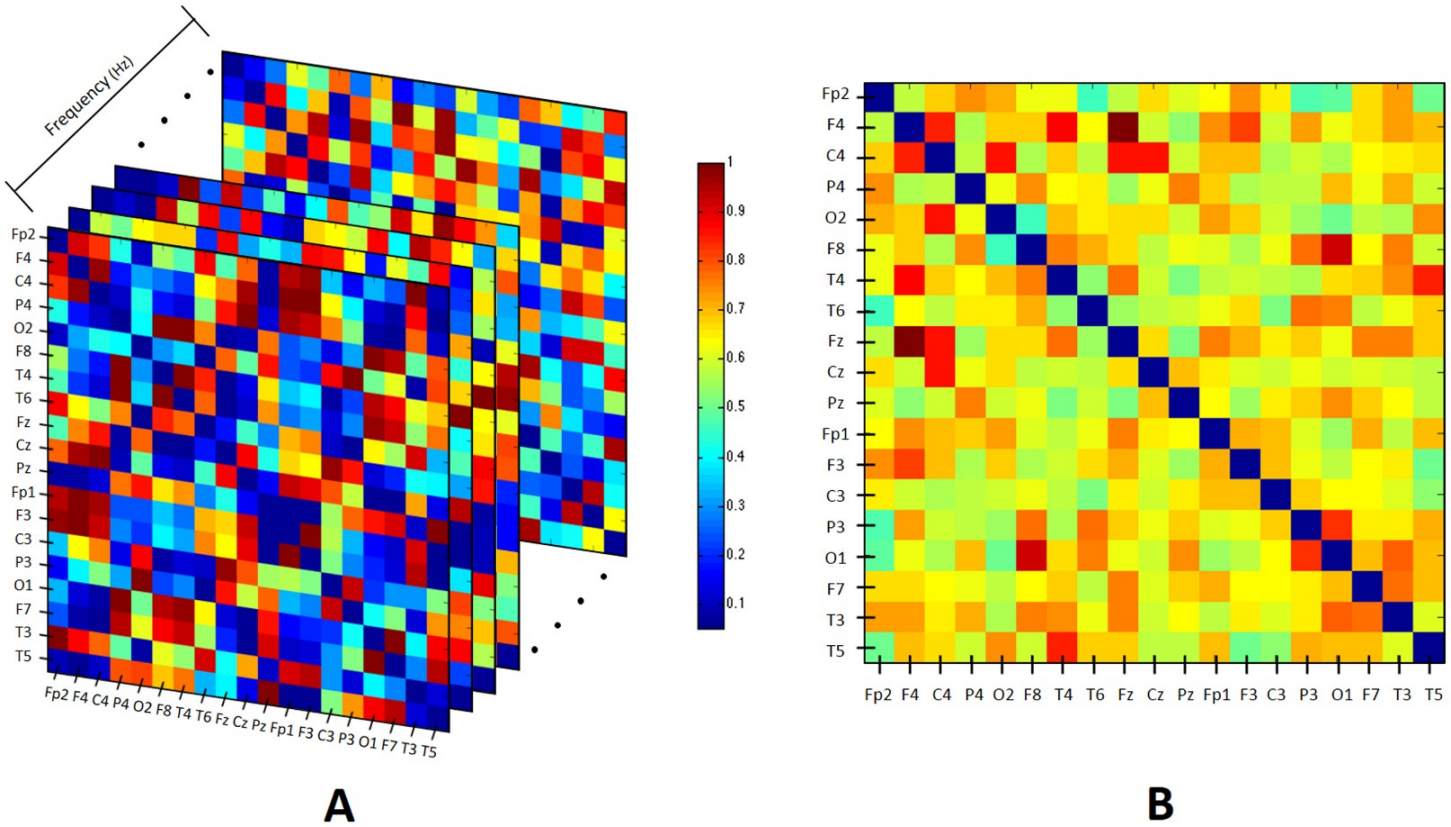
An example of the coherence spectrum matrix for one HA participant is shown in A, while B depicts the average coherence matrix that resulted from the coherence spectrum matrix integration from 0.5–30 Hz.

In this study, the coherence correlation measurement among the 19 electrodes was computed for each of the 57 signals processed using the spatial Laplacian filter (see Eq 1). Fig 3A depicts a coherence spectrum matrix obtained from the EEG recordings of an HA subject. Thereafter, because we wish to obtain a single coherence matrix for specific frequency bands, such as Delta (*δ*, 0.5 − 4 Hz), Theta (*θ*, 4 − 8 Hz), Alpha (*α*, 8 − 13 Hz), and Beta (*β*, 13 − 30 Hz), as well as the 0.5 − 30 Hz band, the average coefficients of the coherence matrices for frequency band were calculated by setting the lower and upper limits of the integral to the above frequency bands. In this way, a coherence matrix was obtained for each frequency band.

The coherence matrix of this same HA participant in the 0.5 − 30 Hz frequency band is shown in Fig 3B. Due to the frequency resolution for the coherence analysis (0.7813 Hz), the ranges of the EEG frequency bands are approximate. The latter results in five coefficient coherence symmetric matrices **M** of dimensions 19 x19 for each subject. At this point, there are 285 matrices. Auto-coherence coefficients (diagonal) for each **M** matrix were removed. To calculate the coherence correlation, the magnitude-squared coherence function from the Matlab^®^ toolkit was used.

#### Thresholding and normalization

Connectivity values often vary among subjects and conditions; that is, most of the coefficients in the **M** matrices are not significant (i.e., they represent very weak interrelationships). Setting a threshold value allowed us to focus only on the relevant connections, ignoring the weak interrelationships. To this end, we defined the threshold and normalization with respect to the minimum and maximum values of the average coherence matrix in order to standardize the metrics for each subject. This led us to propose the threshold equation shown below (3):

$$\begin{array}{c} U=min\left(m_{ij}\right)+\frac{\left(max\left(m_{ij}\right)-min\left(m_{ij}\right)\right)}{2}, \end{array}$$ (3)

where *U* is the threshold value and *min*(*m*_*ij*_) ∈ **M** and *max*(*m*_*ij*_) ∈ **M** are the minimum and maximum values of the non-diagonal elements of the coherence matrix. The resulting matrices were normalized to the maximum value in order to obtain coefficient values between 0 and 1, resulting in a 19 × 19 normalized averaged coherence matrix, which was renamed as the connectivity matrix, per frequency band and for each subject. These matrices are presented in S2 File.

There are some facts to know about before proposing a threshold for coherence values in EEG:

- The coherence coefficient is frequency-band dependent; in other words, the coherence coefficient value for every single frequency reflects the ratio of the common signal registered at two electrode locations, relative to some measure of the total EEG power spectrum (signal plus noise) at the same frequency [46, 47].
- Coherence coefficient values usually span the entire [0, 1] range and decrease systematically as a function of the distance between the electrodes. In other words, one cannot simply consider a value of 0.75 as “high”, or a value of 0.4 as “low” [47–49].

Of course, other non-physiological factors can affect coherence coefficient values; such as the choice of the reference electrode location [47, 50]. However, they depend primarily on the frequency band and the electrode locations. As an example, we can say that coherence values between left and right occipital brain regions above 0.7 are normal for the *α* band, while a coherence value of 0.3 would be normal for the EEG *β* band for the same brain regions [47].

**Fig 4:**
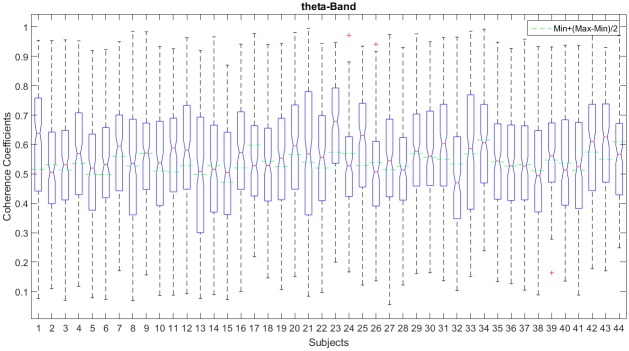
An example of the coherence coefficients distribution (CCD) by subject in *θ* band. The boxplots per subject indicate the 2nd and 3rd quartiles (blue box) which contain 50% of the sample, whiskers contain the 25% of lower and higher coherence values of the sample, the red line indicates the median value, and the green line represents the threshold which lies mostly in the 2nd and 3rd quartiles, taking into account more or less the 50% of the coherence values sample.

In this context, we have analyzed the coherence coefficients distribution for all subjects and bands. Fig 4. shows the boxplots of the Coherence Coefficient Distribution (CCD) by subject, in *θ* band. The dashed green lines indicate the proposed threshold which is near the median (red line) and, in the majority of the subjects it lies under it, letting pass more than the 50% (part of the 2nd, the 3rd and 4th quartiles) of coherence values considered high respect to the median. It is possible to observe that CCD is almost symmetric around the median for most of the subjects. This example shows that most of the subjects’ coherence values range around 0.3 to 0.8 and that the presence of outliers is low in this frequency band. As expected, variability between subjects is high and a dynamic threshold must be chosen.

**Fig 5:**
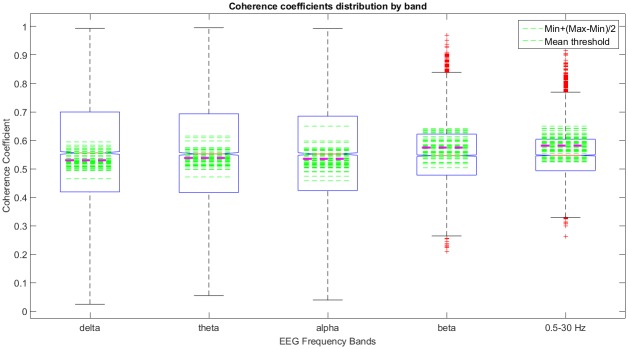
An example of the coherence coefficients distribution in all frequency bands. The green lines represent the proposed threshold per subject, which lie inside the 2nd and 3rd quartiles for δ, θ and α frequency bands, taking into account more or less the 50% of the coherence values sample. For *β* and 0.5 − 30 Hz frequency bands there are some thresholds outside the blue box which lie inside the 4th quartile taking into account less than the 25% of the coherence values sample. The magenta dashed line indicates the mean value of thresholds among the subjects.

From a global point of view, the CCD for all subjects was symmetric for all frequency bands studied here and with symmetry around the median value; however for *β* and 0.5 − 30 Hz frequency bands (the wider bands) we observed the presence of outliers for higher and lower coherence values. This behavior is expected due to the coherence value frequency dependency [46, 47]. The more frequency content of the band, the larger the variability in their coherence values (see Fig 5). We observe also in green dashed lines how the proposed threshold was distributed around the coherence coefficients values for the frequency bands studied here. Clearly, the proposed dynamic thresholds for subject (dashed green lines) are around the median and inside the 50% of the CCD for *δ*, *θ* and *α* frequency bands. However, is not the case for *β* and 0.5 − 30 Hz frequency bands where some of the proposed dynamic thresholds for subject are above the 3rd quartil mainly due to “outliers” presented in those bands. We also observe, in magenta, the mean threshold value among the subjects per frequency band, which lies under the median (2nd quartile) of the CCD for *δ*, *θ*, and *α* frequency bands and, above the median (3rd quartile) for *β* and 0.5 − 30 Hz frequency bands.

The proposed dynamic threshold Eq (3) allows to keep a great number of coherence values considered as high in terms of the CCD. This is true for *δ*, *θ* and *α* frequency bands. In the case of *β* and 0.5 − 30 Hz frequency bands, the proposed threshold is higher than the median value and keeps higher coherence coefficients including those considered as “outliers” for the CCD. Note that *β* and 0.5 − 30 Hz frequency bands have more frequency content than *δ*, *θ* and *α* frequency bands, thus the variability in coherence values is higher producing spurious outliers which are took into account by the proposed threshold.

### Machine-learning processing

In this stage, the data was processed and formatted to fulfill the input requirements of the machine-learning algorithms. We then proceeded to evaluate the proposed features by applying a decision tree algorithm.

#### Feature extraction

At this point, it became possible to represent each participant in terms of her/his brain connectivity using the coherence matrices described in the previous section. To understand changes in brain connectivity, we employed three different graph metrics: degree, edges between neighbors, and the clustering coefficient.

A graph is an ordered triad (*V*, *E*, Ψ) consisting of a non-empty set *V* of vertices, a set of edges *E*, and an incidence function Ψ: *E* → *V*. For each edge, the following condition holds: Ψ associates the edge *e* to a pair of vertices, *V*, Ψ(*e*) = {*u*, *v*}.

The degree of a vertex *d*_*v*_ is the number of connections that vertex *v* has to other vertices, when we consider an undirected network. The clustering coefficient, *C*_*v*_, of the vertex *v* is calculated by Eq (4):

$$\begin{array}{c} C_{v}=\frac{2E_{v}}{\left(d_{v}\right)\left(d_{v}-1\right)}, \end{array}$$ (4)

where *E*_*v*_ is the number of edges between the neighbors of *v*. The clustering coefficient, *C*_*v*_, is not a property of the vertex *v* itself, but a property of its neighbors and *it is a measure of segregation that could be interpreted as: the higher clustering coefficient, the higher the density* [51]. The *C*_*v*_ is related to the local cohesiveness of a network and measures the probability that two vertices with a common neighbor are connected. A highly connected vertex with a low clustering coefficient indicates at least a locally hierarchical structure. On the other hand, many graphs exhibit a rather high clustering, indicating a local cohesiveness and tendency of vertices to form clusters.

While these metrics are mutually highly-correlated, each one provides distinct information to determine changes in brain connectivity: degree provides information on direct connections; edges explain how neighbors are interconnected; and the clustering coefficient reflects the density of a cluster, if it is high, the neighbors are densely interconnected and they are likely to share specialized information [52].

Because of the symmetry of the matrices obtained in the thresholding and normalization section, only 171 of the 361 coherence coefficients were selected (i.e., the upper side of the matrices). Each graph metric produced a column vector of dimensions 1 × 19. As a result, three different column vectors were calculated from each coherence matrix. These three feature vectors will feed the machine-learning algorithm to represent each participant in three distinct feature spaces, each one with a particular meaning in relation to the node connections. It is important to remember that these features are obtained for each one of the aforementioned frequency bands: *δ*, *θ*, *α*, *β*, and the 0.5 − 30 Hz band.

#### Data formatting

From this point forward, our proposal required a reorganization of the data in order to gather each participant’s brain connectivity network information in terms of a given graph metric (*G*) and a specific frequency band (*B*). Expression (5) represents the 15 combinations among the frequency bands and graph metrics. Table 2 presents an example of the structure of the new datasets.

$$\begin{array}{c} B_{i}G_{j}\;\forall i\in \left\{\delta ,\theta ,\alpha ,\beta ,\left(0.5-30Hz\right)\right\},\;\forall j\in \left\{d_{v},E_{v},C_{v}\right\}, \end{array}$$ (5)

**Table 2:** Dataset with the values corresponding to the *d*_*v*_ graph metric, computed on the *α* frequency band, for all participants. The nomenclature to identify this dataset is $B_{\alpha }G_{d_{v}}$ dataset.

Participant	Fp2	F4	…	T5	Achievement level
1	9.10	7.66	…	7.68	HA
2	1.40	8.47	…	8.25	AA
3	3.42	5.79	…	4.53	LA
.	.	.	.	.	.
.	.	.	.	.	.
.	.	.	.	.	.
57	9.83	8.72	…	11.26	LA

These new datasets can be seen as a matrix in which each participant is represented by a row, the columns show the electrodes, and the intersection of both (i.e. a component of the matrix) stores one of the graph metrics calculated from the coherence matrix. Finally, an extra column was added at the right end of the matrix to indicate the achievement level to which each participant belonged. For example, the $B_{\alpha }G_{d_{v}}$ combination corresponds to a dataset that contains the degree of vertex (*d*_*v*_) of the participants calculated over the *α* frequency band. This step was also self-implemented using Matlab^®^.

#### Outliers detection

The metric Silhouette helps interpret the cohesiveness of clusters [53] in a distance-based clustering analysis [54, 55] by assigning a score in the range [−1, 1] to each subject in the predefined cluster. These scores tend to approach 1 when the cluster is cohesive, but tend towards zero when the elements are almost equidistant from two different clusters. Values near −1 suggest that at the average distance the element should be grouped in a different cluster. The procedure used to calculate the Silhouette score is as follows:

Given a set of participants **S** = {*S*_1_, *S*_2_, …, *S*_*n*_}, and several cluster sets **K** = {*K*_1_, *K*_2_, …, *K*_*m*_}, where each *K*_*i*_ = {*S*_*k*_ ∈ *S* ∣ *K*_*i*_ ∩ *K*_*j*_ = ∅, ∀*j* ≠ *i*} with *m*, *n* ∈ *Z*^+^, and where *m* represents the number of clusters, *n* the number of participants, and *i*, *j*, *k*, *l* an index that runs as: *i*, *j* ∈ {1, 2, …*m*} and *k*, *l* ∈ {1, 2, …*n*}. Then, the Silhouette coefficient *s*(*S*_*k*_) is computed as in Eq (6):

$$\begin{array}{c} s\left(S_{k}\right)=\frac{b\left(S_{k},i\right)-a\left(S_{k},i\right)}{\max\{a\left(S_{k},i\right),b\left(S_{k},i\right)\}}, \end{array}$$ (6)

where Eq 7 is the mean distance of *S*_*k*_ ∈ *K*_*i*_ to all participants in its own cluster *i*.

$$\begin{array}{c} a\left(S_{k},i\right)=\frac{1}{|K_{i}|-1}\sum_{S_{l}\in K_{i},k\neq j}d\left(S_{k},S_{l}\right), \end{array}$$ (7)

Eq 8 is the mean distance of *S*_*k*_ ∈ *K*_*i*_ to all participants, *S*_*l*_ ∈ *K*_*j*_, in a cluster different from its own *i* ≠ *j* [53],

$$\begin{array}{c} b\left(S_{k},i\right)=\min_{i\neq j}\frac{1}{|K_{j}|}\sum_{S_{l}\in K_{j}}d\left(S_{k},S_{l}\right), \end{array}$$ (8)

while Eq 9 calculates the distance between a pair of participants using Euclidean distance.

$$\begin{array}{c} d\left(S_{k},S_{l}\right)=\sqrt{\sum_{i=0}^{n}{\left(S_{k}\left[i\right]-S_{l}\left[i\right]\right)}^{2}} \end{array}$$ (9)

To create a computational model as accurate as possible to distinguish different functional association patterns between brain regions, it is important to identify and discard potential outliers [56]. If such outliers are not removed, the effect of their contribution in successive analyses could bias the behavioral patterns of each category of children and, therefore, their brain connectivity patterns. Hence, using the datasets obtained in the data-formatting section, it was possible to evaluate the cohesiveness of the WRAT-4 categorization in terms of a brain connectivity network. Thus, the Silhouette coefficient was calculated for each dataset and for each participant, according to Eq 6. In this step, the value of *m* is 3 and of *n* fifty-seven.

Participants were classified as outliers if two conditions were met: first, if the Silhouette coefficient was lower than or equal to 0; and second, if the proportion of the participants with a Silhouette coefficient greater than 0 in its own dataset was above 70%. Thirteen participants met these criteria and were removed from the final sample. The information on participant distribution in this new dataset is presented in Table 3. In implementing this step we utilized Python 3.7 and a Python toolkit for data-processing and machine-learning named scikit-learn, using a function called *silhouette_score* from the package *sklearn.metrics* [36].

**Table 3:** Number of participants organized by achievement level after the process of outlier elimination.

Achievement level	Total	Age range (years)
HA	15	8.6 − 9.9
AA	14	8.5 − 9.9
LA	15	8.3 − 9.9
Total	44	-

#### Definition of the experiments

In order to perform an analysis of each achievement level, we proposed making a pairwise comparison. Instead of building machine-learning models that classify a participant into one of the three different achievement levels (classes), we designed several bi-class experiments that allowed us to identify connectivity patterns between different brain regions. This simplification is based on the idea that the greater the number of classes into which a sample should be segmented, the greater the number of decision boundaries needed, which makes pattern identification more difficult [57–59]. The pairwise combinations are: HA-AA, AA-LA, HA-LA, HA-OA, AA-OA, and LA-OA, where HA, AA, LA and OA stand for high, average, low and ‘other’ achievement, respectively.

Each experiment contained participants’ information organized by their combined achievement level, frequency band and graph metric. The experiments labeled OA (i.e. HA-OA, AA-OA and LA-OA) contain two sets of information: first, all children categorized as the first part of the combination label (i.e. HA, AA, LA); second, the children belonging to the other two categories, in equal proportions. For instance, half of the participants in the experiments labeled HA-OA correspond to the HA category, while the other half pertain to AA and LA. The latter half was selected randomly, but we maintained an equal number of children from each category while, at the same time, matching them with the total of all participants of the HA category. All experiments and their respective identifier names were created using expression (10).

$$\begin{array}{c} \begin{array}{cc} & Co_{k}\_B_{i}\_G_{j} \end{array} \end{array}$$ (10)

where *Co*_*k*_, ∀*k* ∈ {*HA* − *AA*, *HA* − *LA*, *HA* − *OA*, *AA* − *LA*, *AA* − *OA*, *LA* − *OA*} correspond to a pairwise combination of achievement level, *B*_*i*_, ∀*i* ∈ {*δ*, *θ*, *α*, *β*, 0.5 − 30} correspond to the frequency bands, and *G*_*j*_, ∀*j* ∈ {*d*_*v*_, *E*_*v*_, *C*_*v*_} to the graph metrics.

Hence, based on expression (10), it is possible to obtain 90 experiments represented by a dataset, see S3 File. For instance, the combination $Co_{HA-AA}\_B_{\delta }\_G_{d_{v}}$ corresponds to an experiment/dataset that involved 29 participants (15 HA + 14 AA) and 19 electrodes, where each component has the degree of a vertex (*d*_*v*_) of each electrode over the *δ* band. This step was self-implemented using Matlab^®^.

#### Classification and pattern identification

A decision tree is a supervised machine-learning approach that builds a model depicted as a decision tree. The model is built using a top-down greedy search in which the data are partitioned into subsets that contain homogeneous data. Each subset’s homogeneity is measured using the *information gain* metric. At the end of this process, each node in the decision tree represents the splitting criteria of the data, while the leaves (deeper nodes) represent the target value (a.k.a. class) wherein the instances are classified [60, 61]. Finally, a pruning process is performed to decrease the overfitting effect. An advantage of the DT model is that it facilitates this transformation into decision rules, which makes them human-readable [22]. Based on such rules, it may be possible to reach conclusions on data behavior; that is, based on the attributes selected for the data partitioning it is possible to perform inferences about the dynamics of the problem.

In this work, we propose studying the graph metric characterization using the *C*4.5 decision tree [62]. To do so, we use the 90 experiments/datasets defined in Eq 10. Each experiment represents a pairwise classification problem where the class is identified by *Co*_*k*_. For instance, the combination $Co_{HA-AA}\_B_{\delta }\_G_{d_{v}}$ contains information of two different classes, HA and AA. In summary, the target value could be two out of the three possible achievement levels (HA, AA and LA), but they will be analyzed in pairs.

All experiments were performed using Weka 3.8 software, which has an implementation of the *C*4.5 decision tree algorithm (*J*48) [61, 63]. The parameters of the decision tree algorithm were a confidence factor equal to 0.25, a minimum object per leaf equal to 2, number of folds equal to 3, and pruning set to ‘On’.

The pruned decision trees that resulted from the training phase indicate the nodes selected (i.e. electrodes) for evaluation in a particular feature space (i.e. *G*_*j*_), for each frequency band (i.e. *B*_*i*_).

#### Performance evaluation

Evaluating the performance of different classification models is essential when it comes to selecting the model that best partitions a dataset; that is, in this study, the definition of each experiment. In order to do so, it is possible to use a tool called a confusion matrix, as this approach opens the possibility of registering the number of hits and misses of a particular model while solving a pairwise classification problem. The four possible outcomes of the predictions are: *true positive* (TP) and *true negative* (TN), which are correct classifications; and *false positive* (FP) and *false negative* (FN), which are incorrect classifications. Based on these concepts, it is possible to compute the performance rate, as expressed in Eq (11).

$$\begin{array}{c} Accuracy=\frac{TP+TN}{TP+TN+FP+FN}, \end{array}$$ (11)

Since the datasets are small (44 participants), a stratified, 10-fold cross-validation methodology was applied during experimentation to ensure the reliability of results. This methodology was applied 10 times to add variability.

## Results

The 90 experiments defined in Eq 10 were used as an entrance to the DT algorithm. Table 4 shows the best result for each combination of achievement levels. The first three columns indicate the experiment information (graph metric, frequency band, and combination of achievement levels), whereas the *Accuracy* column indicates the model’s performance.

**Table 4:** Accuracy for the best result of the six achievement level combinations.

Dataset information			Accuracy
Graph metric	Frequency band	Achievement level combination	%
*C*_*v*_	*α*	HA-LA	80.00
*E*_*v*_	*δ*	AA-OA	78.33
*C*_*v*_	*θ*	AA-LA	76.67
*C*_*v*_	*δ*	HA-OA	75.00
*d*_*v*_	0.5 − 30 Hz	LA-OA	74.17
*C*_*v*_	*δ*	HA-AA	68.33

The best accuracy (80.00%) for the DT model corresponds to the prediction of HA vs. LA using the *C*_*v*_ descriptor, analyzed over the *α* frequency band. With respect to the achievement level combinations, five out of six were successfully-classified with an accuracy score above 70% using a DT model; only the combination HA vs. AA had an accuracy score below that threshold. This suggests that the network-based characterization proposed in this work performs well when representing the brain connectivity patterns used to identify the children’s levels of mathematical achievement.

Our results indicate that by observing a specific band and graph metric it is possible to differentiate HA, AA and LA from OA with accuracies of 75.00%, 78.33% and 74.17%, respectively. An important observation is that the participants that are easiest to discriminate from the OA subjects are those in AA, with an accuracy of 78.33%.

With respect to the graph metrics used as features, all of them attained accuracy score greater than 70.00%, suggesting that they were useful for this case of problem representation.

**Fig 6:**
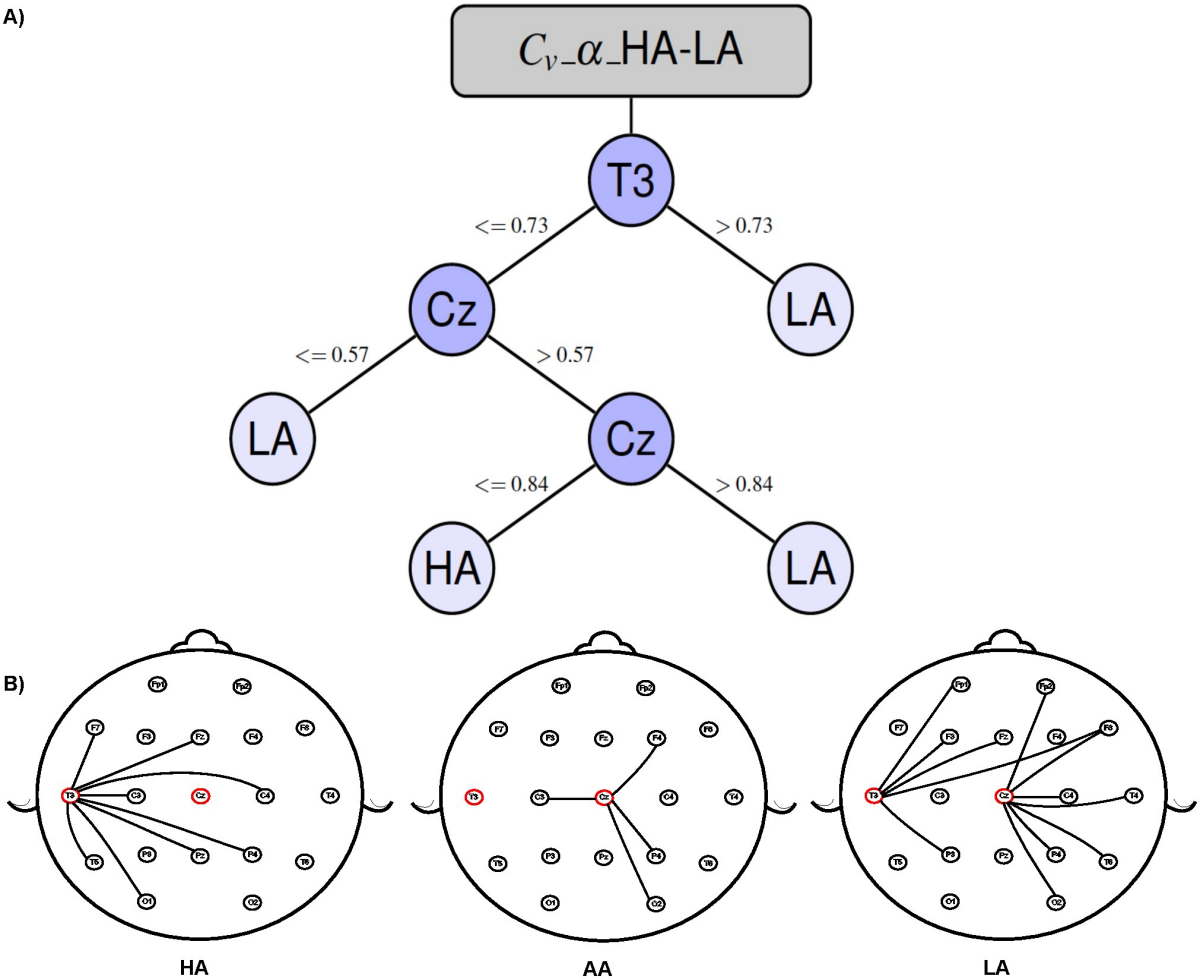
Fig A depicts the decision tree model to classify the high-achievement (HA) level vs. the low-achievement (LA) level for the graph metric *C*_*v*_ on the *α* frequency band with an accuracy of 80%. Fig B depicts the average connectivity behaviour of the electrodes identified by the decision tree model for each achievement level and frequency band.

**Fig 7:**
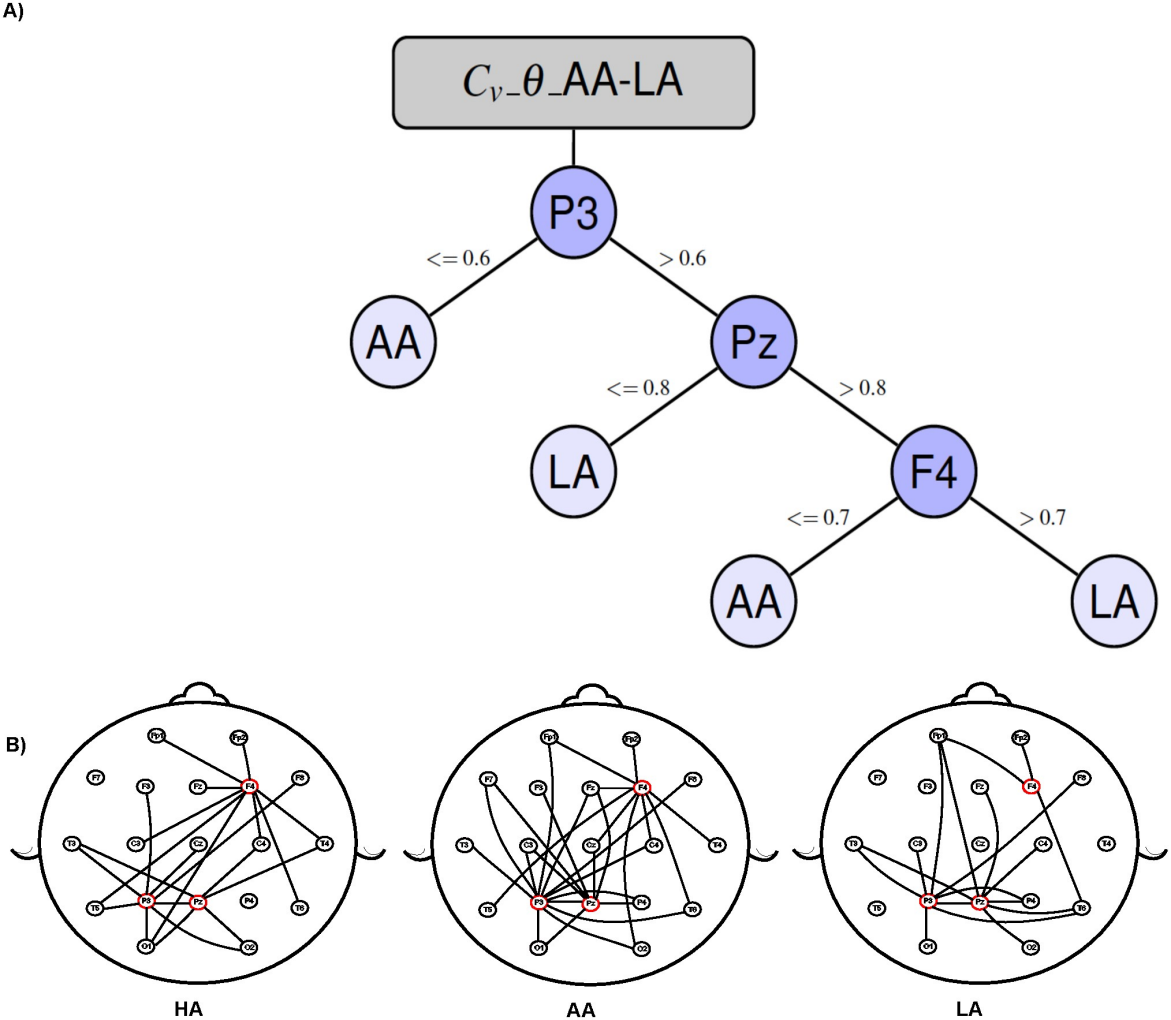
Decision tree model to classify average-achievement (AA) level vs. low-achievement (LA) level for the graph metric *C*_*v*_ on the *θ* frequency band with a 76.77% of accuracy. Fig B depicts the average connectivity behaviour of the electrodes identified by the decision tree model for each achievement level and frequency band.

Regarding the DT models presented in Figs 6 and 7. First, the upper side of the figures shows the DT models built to identify a combination of achievement levels in which the edges are labeled with the thresholds that the calculated electrode metric should fulfill in order to take a path.

Second, the lower side of the images shows the electrode locations on the scalp and the neighborhood of the most important nodes, as identified by the DT model. This neighborhood is obtained from the average of coherence matrices by frequency band and achievement level (HA, LA, and AA). Note that the DT models result from the pattern identification process performed by the DT algorithm when analyzing the participant dataset; in contrast, the graph scheme depicts the average connectivity pattern for a particular frequency band and achievement level.

In this study, therefore, we use the DT models to identify the brain regions that need to be observed to identify electrode connectivity differences between two different classes of participants. For instance, Fig 6A shows that it is possible to differentiate HA from LA in the *α* frequency band by evaluating the clustering coefficient (*C*_*v*_) from electrode recording sites in the left temporal (T3) and middle central (Cz) brain regions. Specifically, a subject will be considered to have a low achievement level if she/he satisfies one of the following conditions: (*T*3 > 0.73) *or* (*T*3 ≤ 0.73 *and Cz* ≤ 0.57) *or* (*T*3 ≤ 0.73 *and Cz* > 0.84); otherwise, the subject will be considered to have a high achievement level.

Fig 7A shows that it is possible to differentiate AA from LA in the *θ* frequency band by evaluating the clustering coefficient (*C*_*v*_) from electrode recording sites placed in the left parietal (P3), middle parietal (Pz) and right frontal (F4) brain regions. Specifically, a subject will be considered with low achievement level if she/he satisfies one of the following conditions: (*P*3 > 0.6) *and* (*Pz* ≤ 0.8) *or* ((*P*3 > 0.6) *and* (*Pz* >= 0.8) *and* (*F*4 > 0.7)) otherwise the subject will be considered with average achievement level.

## Discussion

Previous studies have emphasized the role of the *θ*, *α* and *δ* bands in arithmetic processing and developmental changes. An interesting relationship has been established between *α* activity and dynamic changes of attentional demands during information processing [64]. In reality, when arithmetic tasks are performed, parietal changes in *α* activity seem to be related to the attention network’s working memory function [65]. In this context, it is not surprising that the best accuracy in the predictions made for HA vs. LA by the DT model was achieved while analyzing the *α* frequency band (see Fig 6B). A recent study related an increase in parietal *α* power with autonomic access to long-term memory and a release of attentional resources [66]. Accordingly, our results might be explained as an effect of inter-individual differences in the ability to focus on the task and/or in working memory efficiency.

Another observation was that the electrode recording sites placed over the left temporal and middle central brain regions of the scalp better differentiated the HA and LA groups. Here, the left temporal electrodes might be conveying the effects of functional brain changes from the vicinity of the left angular gyrus, closely-associated with the processing of symbolic numbers [67–69] and the retrieval of arithmetic facts from memory [70, 71], as well as from the inferior temporal gyrus, which seems to be part of a number-processing network [72]. In addition, the middle parietal area might be functionally reflecting activity from the intraparietal sulcus (IPS), a brain region whose impairment has been strongly-related to difficulties in math learning. [73, 74]

A recent experimental study interpreted variations in the *θ* band as evidence of the pre-attentive processing of numerical visual information [75]. Moreover, increases in *θ* power have been reported as an effect of training in arithmetical learning, probably reflecting an improvement in automated procedural and retrieval strategies that lead to a more efficient arithmetic performance [76]. In this context, it has been postulated that activation in frontal areas and the *θ* band data jointly indicate additional domain-general cognitive control and working memory demands for heightened arithmetic complexity in children [77] (see Fig 7B).

In fact, the left parietal, middle parietal and right frontal electrodes better distinguish between AA and LA children. This is consistent with previous developmental imaging studies, which have documented that brain activation during numerical processing shifts from the frontal cortex to the parietal cortex as development progresses [78–80]. A quantitative meta-analysis study also supports the notion that the frontal cortex is important for number-processing in adults [81] and that both regions seem to underlie arithmetic competence [82].

González-Garrido and colleagues reported that middle-childhood individuals with lower arithmetic skills showed significantly higher coherence in the *θ* and *α* frequency bands than their more-skilled peers [11]. The interpretation of these findings coincides with what other authors have reported [83, 84] by acknowledging a major role of the *β* band in numerical-processing, and further suggesting that the number-processing specialization system likely involves several complex, interacting neural networks that require the selection of a cognitive strategy better-suited to individual resource availability. This complexity demands further exploration of these neurofunctional interactions and their behavioral outcomes during neurodevelopment.

Also, results show that the classifier performance was higher for lower frequency bands such as *δ*, *θ*, and *α*. Performance in *β* and 0.5 − 30 Hz. was not so high, probably, due to the coherence coefficient frequency dependence [46, 47] in wider frequency bands. In this context it will be useful to perform a sub-band analysis in future studies, with the aim to refine the resolution to evaluate the frequency bands of interest.

With respect to the graph metrics used as features, the authors of [51] highlighted the importance of the clustering coefficient to quantify the presence of clusters within a network, since the functional segregation in the brain corresponds to the brain ability for specialized processing within densely interconnected brain regions. In addition, [85] mentioned that the clustering coefficient in network neuroscience is important for understanding function-structure associations in the brain because of the abundance of connected triangles around a given node inform us about functions and structures in the brain. This is consistent with our results, considering that, even though all the metrics are useful for classifying different levels of mathematical achievement, the clustering coefficient is the one that appears the most.

Some earlier studies are related to predicting different mental mathematical tasks [13–16]. All those works share a common workflow that involves the pre-processing and characterization of the signal along with the construction of a machine-learning model that captures the correlation that exists between a set of proposed features and a mathematical task performed by a participant or group of subjects. This correlation is expressed by the percentage of accuracy obtained by each proposal. However, some methodological differences may arise when comparing our work with state-of-the-art research.

As in [13, 14], our work also proposes a set of features that capture the dynamics of the brain network. Specifically, both approaches sought to establish neural synchronization patterns of a type of response (correct and incorrect) during the solving of math problems. The authors of [13] proposed using the correlation coefficient (Corr) and phase-locking value (PLV), while the authors of [14] used power spectra (PS), PLV, the directed phase-lag index (dPLI), phase-to-amplitude cross-frequency coupling (PAC), and delay symbolic transfer entropy (dSTE). In this work, we proposed using three graph metrics *C*_*v*_, *E*_*v*_, *d*_*v*_ to characterize brain network dynamics.

With respect to [13], we find that their methodology and ours found that important connectivity patterns arise while a mathematical task is executed by analyzing the *α* frequency band. In addition, our proposal found patterns in the *θ* and *δ* frequency bands, which have previously been reported as important during arithmetic and developmental processes [64]. Also, considering that these two methodologies use distinct electrode position conventions (10 − 10 vs. 10 − 20), two electrodes considered important by [13], C1 and C5, are close to those presented in this proposal, *Cz* and *T*3. In this sense, while in [13] the most important electrodes are determined by the electrode intersection identified by Corr and PLV; in our study, the electrodes involved in the task are determined by the connectivity patterns found by the DT model and depicted by the decision tree. They reported a 68.6% of predictive accuracy using a SVM, which is a more robust machine-learning model while, in contrast, our proposal achieved an accuracy of 80.00%.

With regard to [14], those authors set out to establish a causal interaction between frontal and parieto-occipital brain regions during performance of a mental mathematical task (addition). Their experiments were split into five different levels (one-to-five) with the fifth being the most difficult. They found that four-out-of-five estimators present accuracy percentages between 53.1 and 69.4%, which are lower than those reported in the present work. The authors of [14] reported 100% accuracy using the dSTE estimator, which is greater than our 80%; however, their proposal tested individually (intra-participant), whereas our proposal considered all participants (cross-participants). The intra-participant approach does not reflect the connectivity patterns found in a group of subjects, which was one of the main objectives of our research. In accordance with the conclusions presented in [14], DT models show that in order to differentiate among participants who belong to different achievement levels –especially between AA and LA– it is important to analyze frontal (*F*4) and parietal (*P*3 and *Pz*) brain regions in the *θ* frequency band.

In contrast, the authors of [15, 16] used several signal metrics to represent EEG signals and determine the existence of correlations between those features and a specific mathematical task. In the first case [15], the estimators used were: power spectrum density (PSD), the auto-regressive mode (AR), six statistical features, and a fractal dimension value (i.e., Higuchi method). The best accuracy they reported was 97.87%, using all features and a SVM model as a classifier. Unlike our proposal, the training and testing phases of the machine-learning model were performed in an intra-participant manner. Also, to determine the importance of each channel during the mental arithmetic task classification, each channel was ranked. The result was that the four most important channels were *F*8, *F*3, *AF*3, *O*2. These findings coincide with the information presented herein, where the frontal and parietal-occipital regions play an important role during performance of a mathematical task. It is important to note that in order to identify the importance of each channel during the classification task, the authors of [15] needed an alternative tool to rank each channel, while in our proposal this is calculated during the training model and depicted by the DT.

Finally, the authors of [16] sought to predict the cognitive workload induced by the difficulty of arithmetic problems, primarily in three different levels: low, medium and high. To do so, the authors computed the power spectra at each frequency bin and used them as features for the classification training phase. The machine-learning model used was linear ridge regression, and it was trained and tested using the information from all participants (cross-participants) and individual subjects (intra-participant). For their cross-participant experimentation, they reported a classification accuracy of 64.75%. In order to identify the most important frequency bands and electrodes, those authors calculated the squared correlation coefficient between the power spectra of each electrode, in each band, in relation to the difficulty of the arithmetic operations, denoted as the Q-value. Both studies (theirs and ours) classify participants in three different classes, but in this respect our proposal presents better performance (80%). Also, our training phase was performed in a cross-participant manner. Finally, they found an EEG band power signature from parietal electrodes (*P*3, *P*4, and *Pz*) when analyzing the *θ* and *α* bands. In this regard, both proposals coincide with ours in that *Pz* and *P*3, along with the *θ* and *α* frequency bands, play an important role in the classification task.

## Conclusion

The network-based connectivity analysis of EEGs makes it possible to distinguish among children with low, average and high mathematical achievement, and to identify several brain regions that are involved operationally in the performance of a numerical-comparison task. The quantitative study of the EEG signals also allowed us to evaluate the brain areas included in a brain network related to math performance.

As a contribution, we present a procedure to identify brain connectivity patterns from EEG signals. To do so, we proposed the use of three graph metrics, namely *E*_*v*_, *C*_*v*_, *d*_*v*_, as features. So, we use a non-linear machine-learning algorithm, known as Decision Tree, that presents a prediction accuracy percentage (70 to 80) as good as or better than those using an SVM [13, 15] or a dSTE estimator [14]. In addition, the DT model, as difference as SVM and dSTE estimator, provides not only the capability of using the resulting model as a predictor for future children’s achievement level but also to identify the electrodes involved in the children’s classification which in previous work were obtained with additional processing like electrodes intersection between Corr and PLV [13].

The current proposal may well be useful for preliminary evaluations of the acquisition of math skills, as part of a general quest to improve the design of specific interventions designed to deal with potential difficulties in math learning. Moreover, it could be helpful in terms of identifying and differentiating brain activity during different tasks and subject conditions.

The *δ* and the extended band (0.5 − 30) Hz frequency bands were in which a distinction among the three achievement levels (HA, AA, LA) was observed.

Our proposal to use graph metrics and DT models outperformed previous studies on mathematical task classification in cross-participant experimentation. Our results are not directly comparable to those of intra-participant experiments since we did not perform experiments of that kind. The DT model’s depiction gives information on the connectivity patterns found in a group of participants, provides a set of rules for classifying subjects, and identifies the electrodes (brain regions) that are important for classifying participants in specific levels of mathematical achievement.

An important limitation of this study is that we did not include the sex of the participants as a variable in our data analysis. Even though [86] it has been suggested recently that sex is not a relevant variable for the development of math skills, we consider that analyzing levels of math achievement according to gender merits further research efforts.

## Supporting information

S1 File Raw EEG signals. This file contains the information on the 57 participants processed, from the raw signal to artifact removal steps.
(ZIP)

S2 File Normalized and thresholded coherence matrices. Contains the information on the 57 participants processed from the “Artifact removal” step to the “Thresholding and normalization” step, for each frequency band (*α*, *β*, *δ*, *θ*, 0.5 − 30 Hz.).
(ZIP)

S3 File Participant features given by graph metric, frequency band and achievement-level combination. This file contains the information obtained from the machine-learning stage, from “Feature extraction” to “Dataset construction”.
(ZIP)
